# In-depth exploration of sugammadex and novel neuromuscular blockade antagonists: a narrative review

**DOI:** 10.3389/fphar.2026.1881882

**Published:** 2026-09-15

**Authors:** Yingbei Qi, Xuan Xu, Xinmin Wu, Lvhong Luo, Qing Jie, Youmao Qi

**Affiliations:** 1 Hangzhou Adamerck Pharmlabs Inc., Hangzhou, China; 2 Department of Anesthesiology, First Hospital, Beijing University, Beijing, China

**Keywords:** adverse events, neuromuscular blockade, neuromuscular blockade reversal, rocuronium, sugammadex

## Abstract

Sugammadex is a modified γ-cyclodextrin widely used in clinical practice to reverse neuromuscular blockade induced by non-depolarizing neuromuscular blockade agents, notably rocuronium and vecuronium. It has been proven to selectively reverse rocuronium-induced neuromuscular blockade within 3 min of administration, representing a revolutionary breakthrough in anesthesiology. Although severe adverse events have been observed in a very small subset of patients, these events have not compromised its overall clinical utility or widespread adoption. This review synthesizes key aspects of sugammadex, including its mechanism of action, pharmacokinetic profile in both healthy and special populations, dosing recommendations for various degrees of neuromuscular blockade in both healthy and special populations, the incidence of adverse effects, pharmacoeconomic considerations, and recent advances in novel reversal agents.

## 1 Introduction

Neuromuscular blocking agents (NMBAs) are commonly employed for surgical interventions to facilitate endotracheal intubation and optimize surgical conditions by suppressing voluntary or reflex muscle movements (Cook and Simons, 2026). After surgery, acetylcholinesterase (AChE) inhibitors such as neostigmine and pyridostigmine have been traditionally used to accelerate the reversal of neuromuscular blockade induced by NMBAs (Neely et al., 2026; Miller, 2007). AChE inhibitors increase acetylcholine availability at the neuromuscular juncture to compete with NMBAs, which can lead to accelerated recovery of muscle function. However, AChE inhibitors are prone to ceiling effects and are unable to reverse deep neuromuscular blockade induced by NMBAs. In addition, AChE inhibitors can potentially increase the risk of residual neuromuscular blockade, and the recovery speed of neuromuscular function is unpredictable (Bartels and Hunter, 2020; Kuo et al., 2022; Thilen et al., 2023).

In 2008 and 2015, a novel first-in-class drug known as sugammadex was approved in the European Medicines Agency (EMA) and US Food and Drug Administration (FDA), respectively, for the reversal of rocuronium- and vecuronium-induced neuromuscular blockade (Figure 1) (Keating, 2016; Merck Sharp and Dohme LLC, 2026). Sugammadex is a modified γ-cyclodextrin with an encapsulation mechanism that can encapsulate NMBAs such as rocuronium and vecuronium, preventing further neuromuscular blocking and elimination through the urine and showing strong efficacy in reversing moderate and deep neuromuscular blockade (Figure 2) (Chandrasekhar et al., 2026). Potential adverse events of sugammadex, including hypersensitivity, bradycardia, a prolonged QT interval, and alterations in coagulation function, have been reported in a very small subset of patients after the approval market (Arslan et al., 2021; Teng et al., 2021; Lee, 2019). Pharmacoeconomic analyses suggest that, despite its high unit price, sugammadex may be cost-effective in certain clinical settings (Paton et al., 2010; Carron et al., 2016). Given its extensive use, an in-depth evaluation of sugammadex remains warranted. This review summarizes its pharmacological properties, clinical safety profile, and pharmacoeconomic considerations while also offering perspectives on the development of novel reversal agents.

**FIGURE 1:**
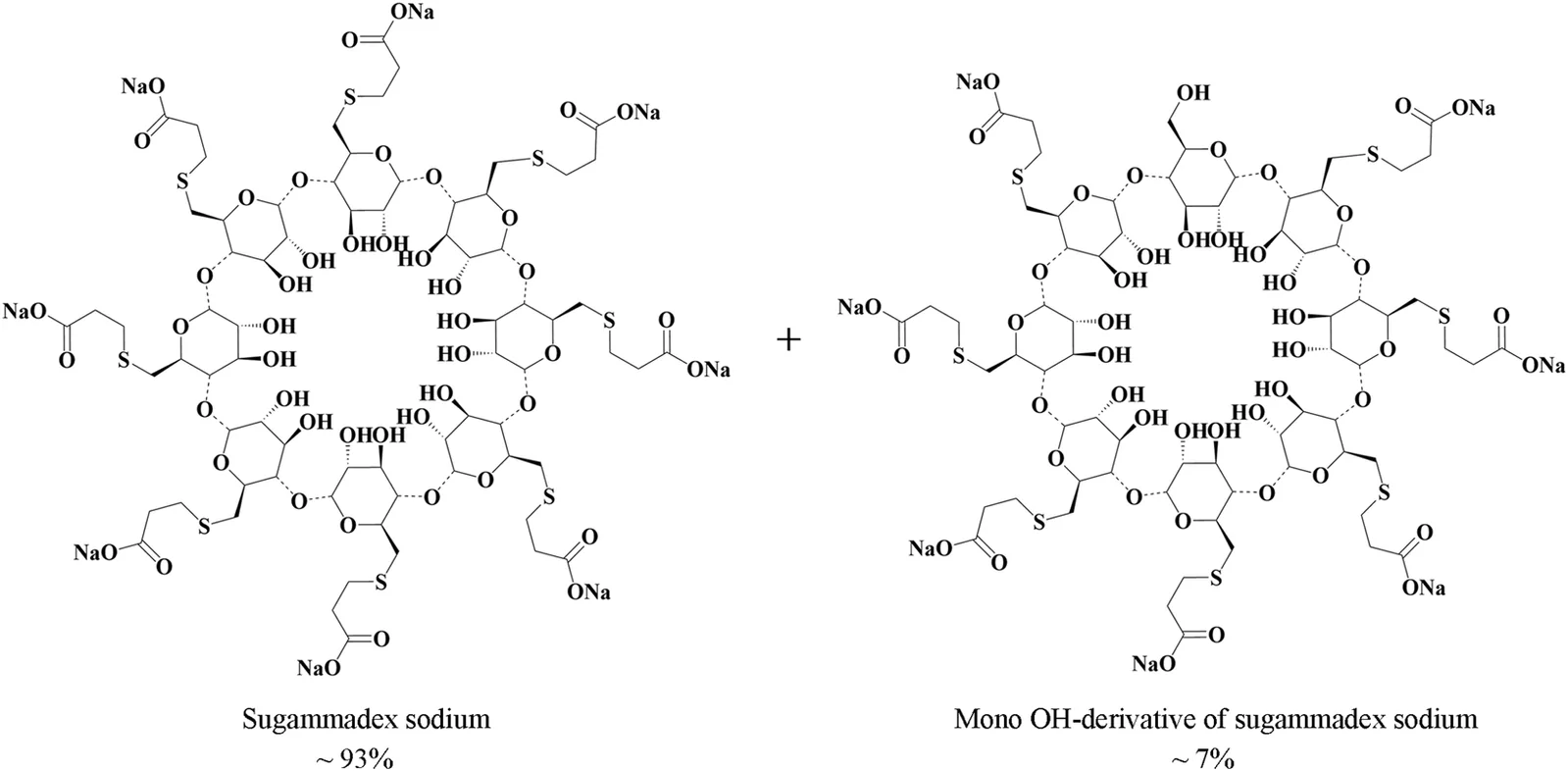
Chemical structure diagram showing sugammadex sodium with eight side chains modified with 3-mercaptopropionic acid on the left, comprising approximately ninety-three percent, and a mono OH-derivative of sugammadex sodium on the right, comprising approximately seven percent.

**FIGURE 2:**
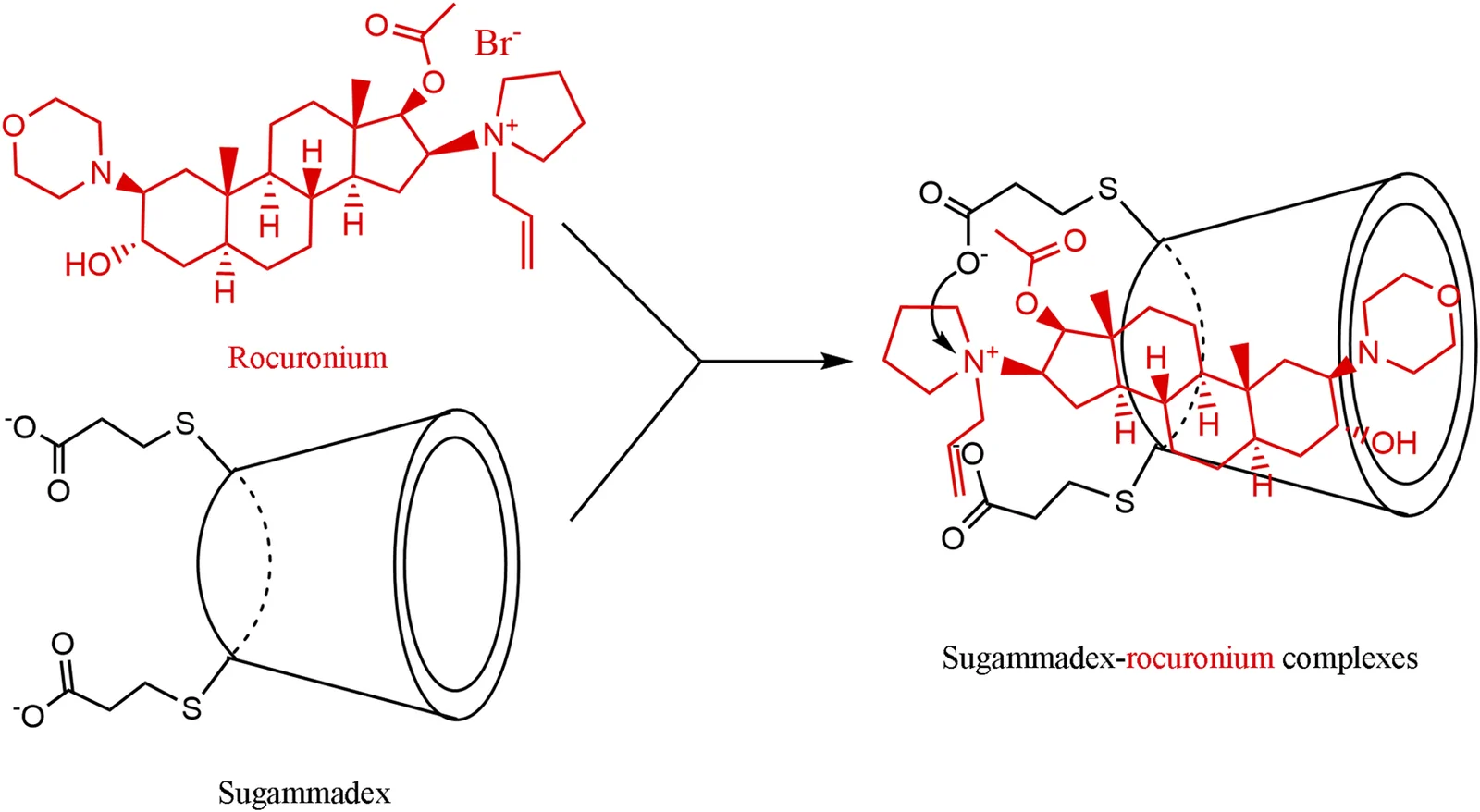
Chemical diagram showing the molecular structures of rocuronium and sugammadex, with arrows indicating their combination to form a sugammadex-rocuronium complex, where rocuronium is encapsulated within the sugammadex ring.

## 2 Methods

We performed a literature search using the PubMed and US FDA drug databases to identify English language publications. The following search terms were used: sugammadex and pharmacokinetics; sugammadex and therapeutic efficacy; sugammadex and residual neuromuscular blockade; sugammadex and recurarization; sugammadex and hypersensitivity; sugammadex and anaphylaxis; sugammadex and coagulation; sugammadex and cardiovascular effects; neuromuscular blockade; neostigmine; adamgammadex; calabadions. After reviewing the abstracts of the screened articles, the final selection was based on their direct relevance to the topics, with a focus on the most recent and salient evidence. The protocol for this narrative review was registered with the International Prospective Register of Systematic Reviews (PROSPERO; registration number: CRD420261296206; website: https://www.crd.york.ac.uk/PROSPERO/view/CRD420261296206).

## 3 Neuropharmacology of sugammadex

The neuropharmacological profile of sugammadex is characterized by its unique mechanism of action at the neuromuscular junction, which differs fundamentally from that of conventional reversal agents (Nicholson et al., 2007). Mechanistically, sugammadex does not directly act at the nicotinic acetylcholine receptor; rather, it acts as a molecular encapsulating agent in the plasma compartment, forming a tight, essentially irreversible 1:1 host-guest complex with rocuronium or vecuronium. This encapsulation rapidly decreases the free plasma concentration of NMBA, creating a concentration gradient that draws NMBA molecules away from the neuromuscular junction and back into the plasma, where they are subsequently encapsulated (Naguib, 2007). As the pool of free NMBAs available to occupy postsynaptic nicotinic acetylcholine receptors diminishes, endogenous acetylcholine released from the presynaptic terminal is able to outcompete residual NMBAs for receptor binding, thereby restoring normal synaptic transmission and neuromuscular function. This receptor-independent, encapsulation-based mechanism is fundamentally distinct from that of AChE inhibitors such as neostigmine, which act indirectly by increasing the synaptic acetylcholine concentration rather than by removing the antagonist itself (Nicholson et al., 2007; Naguib, 2007). From a broader neuropharmacological perspective, this mechanism illustrates that the selective molecular sequestration of a receptor ligand can be as effective a strategy for reversing pharmacological blockade as direct modulation of receptor-level signaling, highlighting an alternative paradigm in the design of reversal agents for neuromuscular blocking drugs (Rex et al., 2010).

## 4 Pharmacokinetic properties of sugammadex

Sugammadex can achieve rapid selective reversal of rocuronium or vecuronium by encapsulating it in the cavity of cyclodextrin, resulting in the formation of a stable 1:1 complex (Nguyen-Lee et al., 2018). This encapsulation ability reduces the amount of free NMBA available at the neuromuscular junction that is available to bind to nicotinic acetylcholine receptors (Nguyen-Lee et al., 2018).

### 4.1 Pharmacokinetics in healthy populations

In clinical settings, the pharmacokinetic properties of sugammadex have been extensively studied (Peeters et al., 2010; Kam et al., 2015; Cada et al., 2016). The intravenous administration of low doses of sugammadex (1–8 mg/kg) can rapidly reverse the neuromuscular blockade induced by 0.6 mg/kg rocuronium in a linear dose-dependent manner, with 95% of the dose secreted in the urine within 48 h (Gijsenbergh et al., 2005; Peeters et al., 2011). The intravenous administration of high levels of sugammadex (32–96 mg/kg) has linear pharmacokinetics, with 90%–93% of the dose secreted in the urine within 48 h (Peeters et al., 2010). Sugammadex has an observed volume of distribution at a steady state of 11–14 L in adults with normal renal function (Cada et al., 2016). There are no metabolites of sugammadex detected in clinical studies, implying that it is not metabolized in the body and is excreted by the kidneys as an unchanged drug (Keating et al., 2016; Kim et al., 2021). In adult patients with normal renal function, the plasma clearance rate of sugammadex is 88 mL/min, and the half-life is 2 h. The pharmacokinetic profiles of sugammadex in healthy Chinese, Japanese, and Caucasian individuals are similar (Merck Sharp and Dohme LLC, 2026; Yuan et al., 2021). The pharmacokinetic data of sugammadex in healthy subjects after administration from some studies are shown in Table 1.

**TABLE 1:** Pharmacokinetic parameters of sugammadex (Gijsenbergh et al., 2005; Yuan et al., 2021; Kam et al., 2015; Peeters et al., 2012).

Parameters	AUC_0–∞_ (μg·min/mL)	CL (mL/min)	T_1/2_ (min)	T_max_ (min)	C_max_ (μg/mL)
1 mg/kg (n = 11) (Yuan et al., 2021)	652	99	105.8	2.0 (2.0–3.0)	17.1
2 mg/kg (n = 11) (Yuan et al., 2021)	1114	116	96.1	2.0 (2.0–3.0)	31.2
4 mg/kg (n = 11) (Yuan et al., 2021)	2202	116	102.6	2.5 (2.0–3.0)	61.3
8 mg/kg (n = 4) (Gijsenbergh et al., 2005)	4837	122	97.9	—	—
16 mg/kg (n = 12) (Kam et al., 2015)	8971	99.7	145	2.5 (2.0–3.0)	197
32 mg/kg (n = 12) (Peeters et al., 2010)	22301	111	234	7.0 (5.0–10.0)	343
64 mg/kg (n = 12) (Peeters et al., 2010)	46245	107	258	7.0 (5.0–10.0)	648
96 mg/kg (n = 12) (Peeters et al., 2010)	71273	104	186	7.0 (5.0–10.0)	1168

All parameters are presented as arithmetic means at 1, 2 and 4 mg/kg and as geometric means at 8, 16, 32, 64 and 96 mg/kg, except for T_max_, which is the median (range). AUC_0–∞_ = area under the plasma concentration‒time curve from time zero to infinity; CL, total body clearance of the drug from plasma; C_max_ = maximum plasma concentration; T_max_ = time to reach C_max_; T_1/2_ = terminal elimination half-life.

### 4.2 Pharmacokinetics in patients with renal impairment

Clinical studies have shown that the T_1/2_ of sugammadex is prolonged to 4, 6, and 19 h in patients with mild, moderate, and severe renal impairment, respectively (Merck Sharp and Dohme LLC, 2026). A phase 1 pharmacokinetic study demonstrated that 4 mg/kg sugammadex was well tolerated in subjects with moderate renal impairment (creatinine clearance of 30–50 mL/min), with a safety profile similar to that of healthy subjects. In this study, pharmacokinetic data were obtained from 18 subjects; the mean sugammadex exposure (AUC_0–∞_) in subjects with moderate and severe renal impairment was 2.42- and 5.42-fold greater, respectively, than that in healthy controls. The clearance decreased and the apparent terminal half-life was prolonged with increasing renal dysfunction, while the C_max_ values remained similar across groups, and no serious adverse events were reported. Thus, despite increased exposure, no dose adjustment is required for mild to moderate renal impairment, as elevated exposure does not lead to increased safety risks (Min et al., 2017). However, current safety experience is insufficient to support the use of sugammadex in patients with creatinine clearance <30 mL/min; therefore, its use is not recommended in patients with severe renal impairment (Min et al., 2017; Oh and Lim, 2023).

Interestingly, several experimental studies have shown that sugammadex has a nephroprotective effect in rats with renal ischemia-reperfusion injury (Te et al., 2021; Kölükçü et al., 2025). Therefore, whether increased exposure to and accumulation of sugammadex in the body confer clinical benefits or potential safety risks remains unclear. Further clinical studies are needed to provide more evidence.

In a multicenter, parallel-group, comparative, open-label study, patients were stratified by age into adult (18–64 years), elderly (65–74 years), and old-elderly (≥75 years) groups. Pharmacokinetic analysis revealed that sugammadex clearance decreased with increasing age: the clearance was 0.104 L/min (T_1/2_ = 2.4 h) in adults, 0.076 L/min (T_1/2_ = 3.2 h) in the elderly group, and 0.052 L/min (T_1/2_ = 4.6 h) in the old-elderly group. Clearance in the old-elderly group was approximately 50% lower than that in adults, with a corresponding T_1/2_ approximately twice as long, whereas no clinically significant differences in volume of distribution were observed across the three groups. Sugammadex was well tolerated across all age groups, and sugammadex 2.0 mg/kg rapidly reversed rocuronium-induced neuromuscular blockade in all age groups. Although recovery was slightly slower in elderly patients (old-elderly 3.6 min vs. adults 2.3 min), it remained within a rapid range. The reduction in clearance in elderly patients was primarily attributed to an age-related decline in renal function, and no dose adjustment is required based on age (Merck Sharp and Dohme LLC, 2026; McDonagh et al., 2011).

## 5 Therapeutic efficacy of sugammadex

The therapeutic efficacy of sugammadex in reversing the moderate and deep neuromuscular blockade of rocuronium and vecuronium is dependent on the dose administered. This dose dependency is in line with the ability of sugammadex to encapsulate rocuronium. One study revealed that the injection of 2 mg/kg sugammadex during the reappearance of the second twitch (T_2_) of train-of-four (TOF) stimulation resulted in a rapid dose-dependent reversal of the moderate neuromuscular blockade induced by rocuronium and vecuronium (Puhringer et al., 2010). In another study on the reversal of deep neuromuscular blockade, a dose of 4 mg/kg sugammadex was suggested to reverse the TOF ratio (TOFR) to 0.9 within 2 min at the reappearance of 1–2 posttetanic (PTC) counts (Duvaldestin et al., 2010). In outpatient surgery patients, the median time to recovery of TOFR ≥90% was significantly lower in the sugammadex group than in the neostigmine group (180 s vs. 540 s, respectively; *p* = 0.0052) (Fiorda Diaz et al., 2022).

### 5.1 Reversal of moderate neuromuscular blockade

In reversing moderate neuromuscular blockade, randomized, multicenter assessor-blind or double-blind trials comparing the efficacy of sugammadex with that of neostigmine administered at the reappearance of T_2_ for the reversal of moderate neuromuscular blockade have been conducted; in these studies, the geometric mean time to recovery of TOFR to 0.9 was significantly shorter in the sugammadex group than that in the neostigmine group (1.4–1.8 min vs. 6.7–18.6 min, *p* < 0.001 or *p* < 0.0001 in all studies) (Illman et al., 2011; Blobner et al., 2010; Woo et al., 2013; Wu et al., 2014).

In a further clinical study, patients who underwent surgery were given 0.6 mg/kg rocuronium prior to intubation, and sugammadex 2.0 mg/kg or neostigmine 50 μg/kg with glycopyrrolate 10 μg/kg was given to the Korean patients, achieving a TOFR of 0.9 (1.8 min with sugammadex vs. 14.8 min with neostigmine) (Woo et al., 2013). This is similar to that of Caucasian patients, who achieved a TOFR of 0.9 (1.5 min with sugammadex vs. 18.6 min with neostigmine) (Blobner et al., 2010). Studies of Chinese patients also revealed that the TOFRs of patients who received sugammadex and neostigmine, which were 91% and 2%, respectively, reached 0.9 within 3 min (Wu et al., 2014); the detailed results are shown in Table 2.

**TABLE 2:** Sugammadex *versus* neostigmine in the time (min) to recovery of TOFR ≥90% (moderate antagonism) (Illman et al., 2011; Blobner et al., 2010; Woo et al., 2013; Wu et al., 2014).

Parameters	Sugammadex		Neostigmine	
	Geometric mean (95% CI)	Number	Geometric mean (95% CI)	Number
Finns (Illman et al., 2011)	1.7 (0.7–3.5)*	24	13.3 (3.5–28.9)*	23
European (Blobner et al., 2010)	1.5 (1.3–1.6)	47	18.6 (14.2–24.4)	45
Korean (Woo et al., 2013)	1.8 (1.6–2.0)	59	14.8 (12.4–7.6)	59
Chinese (Wu et al., 2014)	1.6 (1.5–1.7)	119	9.1 (8.0–10.3)	111
Caucasian (Wu et al., 2014)	1.4 (1.3–1.5)	29	6.7 (5.5–8.0)	30

* mean (range).

### 5.2 Reversal of deep neuromuscular blockade

Randomized trials have been performed to determine the efficacy of sugammadex for reversing deep neuromuscular blockade (Abola et al., 2020; Togioka et al., 2020; Geldner et al., 2012; Jones et al., 2008; Lemmens et al., 2010; Rahe-Meyer et al., 2015). Patients were intravenously administered 4 mg/kg sugammadex, 50–70 μg/kg neostigmine along with atropine, or a placebo to compare the overall efficacy of rocuronium reversal. The recovery time to a TOFR of ≥0.9 with sugammadex was significantly faster than that with neostigmine (2.2–4.5 min vs. 49.0–89.8 min, *p* < 0.0001 in all studies), detailed results are shown in Table 3 (Jones et al., 2008; Lemmens et al., 2010; Rahe-Meyer et al., 2015).

**TABLE 3:** Sugammadex *versus* neostigmine in the time (min) to recovery of TOFR ≥90% (deep antagonism) (Jones et al., 2008; Lemmens et al., 2010; Rahe-Meyer et al., 2015).

Parameters	Sugammadex		Neostigmine	
	Geometric mean (95% CI)	Number	Geometric mean (95% CI)	Number
American (Jones et al., 2008)	2.7 (2.1–4.1)*	37	49.0 (35.7–65.6)*	37
American (Lemmens et al., 2010)	4.5 (2.3–6.6)*	47	66.2 (46.0–96.6)*	36
American (Rahe-Meyer et al., 2015)	2.2 (1.9–2.5)	69	89.8 (80.1–100.7)	65

* median (interquartile range).

In deep neuromuscular block, sugammadex has demonstrated superiority over neostigmine in reversing deep neuromuscular blockade, as it avoids the limitations of neostigmine, which include the inability to reverse profound blockade, potential muscle weakness, and cardiovascular events (Luo et al., 2018).

### 5.3 Immediate reversal of neuromuscular blockade

A multicenter study evaluated the efficacy of 16 mg/kg sugammadex for immediate reversal administered at 3 min post-rocuronium. The results demonstrated a significantly shorter mean recovery time with sugammadex than with spontaneous recovery after 1 mg/kg succinylcholine: to 10% of T1 (4.4 vs. 7.1 min) and to 90% of T1 (6.2 vs. 10.9 min) (Lee et al., 2009). However, the role of sugammadex in managing “cannot intubate, cannot ventilate” (CICV) scenarios remains controversial. A systematic review of cases with a median (range) dosage of 14 (5–16) mg/kg sugammadex, where CICV occurred minutes after induction, revealed the restoration of adequate spontaneous ventilation in 6/8 (75%) patients; the remaining 2 cases (25%) resulted in an obstructed pattern of breathing and required a surgical airway. This discrepancy reflects the inherent gap between ideal efficacy and real-world effectiveness. In the multicenter study, sugammadex was given at exactly 3 min with a full 16 mg/kg dose, ensuring rapid recovery. In CICV scenarios, administration is often delayed, and dosing is inadequate (median 14 mg/kg, range 5–16). Moreover, anatomical obstruction persists despite neuromuscular reversal, which explains the 25% surgical airway rate. Although these case reports suggest that sugammadex might be helpful in CICV scenarios, further research is needed to confirm its effectiveness. However, due to the rare occurrence of CICV events, gathering sufficient data for conclusive evidence may prove challenging (Abou Nafeh et al., 2024). Therefore, the clinical strategy should be to administer sugammadex as a rescue attempt while simultaneously preparing for surgical airway access; if ventilation does not improve within 1–2 min, the patient should proceed immediately to anterior neck airway access.

### 5.4 Special patient populations for reversal of neuromuscular blockade

#### 5.4.1 Children and infants

Sugammadex use in pediatric patients is common (Faulk et al., 2021). Studies have shown that compared with neostigmine, sugammadex is more effective at reversing neuromuscular blockade in children and neonates without significant adverse events (Gaver et al., 2019; Alonso et al., 2014; Carlos et al., 2016; Lang et al., 2022). Similar to adults, pediatric patients can experience residual neuromuscular blockade with sugammadex if not properly monitored. Cases of postoperative weakness have been reported, particularly in children under 2 years of age, highlighting the critical need for quantitative TOF assessments (Carollo and White, 2019). A large study with nearly 34,000 children revealed that effective monitoring might be more crucial than the choice of reversal agent itself (Beltran et al., 2022). According to established strategies for ensuring adequate oxygenation and ventilation while following updated protocols for pediatric airway management is recommended (Apfelbaum et al., 2022). Quantitative monitoring is strongly recommended, especially when it deviates from the manufacturer’s dosing recommendations (Cha and Faulk, 2022).

#### 5.4.2 Pregnant and lactating women

Current guidelines prioritize the use of neuraxial anesthesia over general anesthesia for cesarean delivery (Stourac et al., 2015). In such cases, rocuronium is administered during induction, even though the induction of muscle relaxation through the use of general anesthesia is often unnecessary because of pregnancy-associated abdominal wall distension and uterine evacuation during cesarean delivery (Richardson and Raymond, 2020). A trial of 240 pregnant patients demonstrated successful and complete reversal of rocuronium-induced neuromuscular blockade with sugammadex, even in cases of deep block, without the need for additional doses (Stourac et al., 2016). Other findings also support sugammadex as an effective and safe option for reversing rocuronium-induced neuromuscular blockade at the end of cesarean delivery, with a typical dosing strategy of 2–4 mg/kg on the basis of neuromuscular response monitoring (Nauheimer et al., 2012; Shibusawa et al., 2012).

Failed tracheal intubation, although rare, remains a significant concern in general anesthesia for cesarean delivery. Traditionally, succinylcholine has been used during rapid sequence induction because of its rapid onset and transient duration, allowing spontaneous ventilation to resume if intubation fails (McDonnell et al., 2008; Kinsella et al., 2015). However, succinylcholine has several side effects, including myalgias, hyperkalemia, and increased intraocular pressure (Hager et al., 2026). Rocuronium, at high doses, offers an alternative with comparable onset speed but without these side effects, although it produces prolonged paralysis and cannot be reversed with neostigmine in a CICV scenario (Naguib et al., 2016). High-dose sugammadex (16 mg/kg) can effectively reverse profound rocuronium-induced neuromuscular blockade, as recommended by some guidelines, despite the limited clinical evidence of its use in actual CICV situations in pregnant patients (Naguib et al., 2016; Pühringer et al., 2008; de Boer et al., 2007; Lee et al., 2009; Kopman and Kurata, 2012; Curtis et al., 2012).

The safety profile of sugammadex during pregnancy, such as potential placental transfer, remains unknown. Although its molecular structure suggests that minimal transfer occurs, no confirming evidence exists (Jahr et al., 2015; Nag et al., 2013). One report noted successful maternal sugammadex use with subsequent fetal rocuronium administration, suggesting that fetal exposure is limited (Munro et al., 2019). The finding that sugammadex induced neuronal apoptosis in preclinical studies raises concerns about its potential developmental effects, although these effects were absent in models with intact blood‒brain barriers or in studies in which neuromuscular blockers were co-administered (Palanca et al., 2013).

With regard to lactation, no controlled human data on the excretion of sugammadex into breast milk are currently available. Preclinical studies have raised concerns: in pregnant rabbits, daily administration of supratherapeutic doses resulted in decreased fetal body weight and incomplete ossification, although these developmental effects were not reproduced in rat models, suggesting potential species differences (Merck Sharp and Dohme LLC, 2026). The Society for Obstetric Anesthesia and Perinatology (SOAP)’s 2019 statement advises counseling patients who the effect of sugammadex on lactation is unknown, while a normal breastfeeding routine can likely be resumed once the mother has recovered from anesthesia; the SOAP statement accordingly recommends traditional reversal agents during pregnancy and the postpartum period. If sugammadex is used, it should be avoided in the first trimester and should be used with caution at or near term for cesarean delivery and in the immediate postpartum period (with patients informed that its effects on lactation are unknown) and that additional non-hormonal contraception should be used for 7 days following its administration in reproductive-age women using hormonal contraceptives (Society for Obstetric Anesthesia and Perinatology, 2019). On the basis of the drug’s large molecular size, high polarity, and low oral bioavailability, other guidelines, including the Drugs and Lactation Database (LactMed) and a 2020 obstetric anesthesia guideline on breastfeeding, consider clinically significant transfer to a breastfed infant unlikely and regard use during breastfeeding as acceptable (Mitchell et al., 2020; National Institute of Child Health and Human Development, 2006). This difference between SOAP and other guidance reflects the relative paucity of direct pharmacokinetic data in breastfeeding women rather than a disagreement over documented harm.

#### 5.4.3 Elderly individuals

Several studies have investigated how aging affects the efficacy and pharmacokinetics of sugammadex in elderly patients. In general, compared with younger patients, patients older than 65 years have a slight prolongation of recovery time, with a typical recovery time prolonged for approximately 1–2 min because of an age-associated decline in renal function (Carron et al., 2018). Despite this minor delay, the recovery rate is still considered clinically acceptable, and no changes to dosing recommendations have been made. However, some debate persists regarding whether the prolonged recovery time in elderly patients may be linked to reduced cardiac output (Yoshida et al., 2012).

#### 5.4.4 Obese patients

In obese patients, a dosing discrepancy exists between rocuronium and sugammadex: rocuronium dosing is typically based on ideal body weight, whereas sugammadex is recommended to be dosed according to actual body weight (Meyhoff et al., 2009; Leykin et al., 2004; Monk et al., 2017). This distinction is clinically important, as sugammadex dosed solely on the basis of ideal body weight has been found to be insufficient to reliably reverse deep and moderate neuromuscular blocks in morbidly obese patients (Llaurado et al., 2012). However, an alternative strategy using ideal body weight plus an additional 40% was effective, with a mean reversal time of less than 2 min (Gulliya et al., 1990). Currently, no dose adjustments of sugammadex are needed for obese patients (Monk et al., 2017). Regardless of the dosing approach, neuromuscular monitoring remains essential in all cases to ensure that the recurrence of neuromuscular blockade does not occur, ensuring that sugammadex dosing is adequate in obese patients.

#### 5.4.5 Patients with renal failure

Sugammadex in patients with renal failure poses unique challenges and considerations. Renal impairment affects the pharmacokinetics of many drugs, and studies have shown that the elimination of sugammadex is significantly altered in this population, leading to a prolonged drug half-life and accumulation (MacIntyre, 2024; Paredes et al., 2020). This can result in an increased risk of residual neuromuscular block and potential adverse events. A comprehensive clinical study evaluated sugammadex in 219 patients with end-stage renal disease, including 143 on hemodialysis and 28 on peritoneal dialysis. Among them, approximately 50 patients (22.3%) experienced postoperative complications, and 9 (4.1%) died within 30 days. Critically, none of these adverse outcomes were determined to be linked to sugammadex. This evidence suggests that sugammadex may be a viable reversal agent in this high-risk population (Paredes et al., 2020). Nevertheless, a recent large-scale propensity-matched study offered a comparative perspective, suggesting that in patients with chronic kidney disease, the choice of neuromuscular blocking agent and its reversal may significantly impact outcomes. This study revealed that the use of rocuronium with sugammadex was associated with a significantly greater risk of respiratory failure, acute respiratory distress, hypertensive crisis, heart failure, pleural effusion, and 30-day mortality than the use of cisatracurium with neostigmine (Georgakis et al., 2025). These findings highlight the importance of considering alternative neuromuscular management strategies in patients with renal impairment and underscore that despite the apparent safety of sugammadex in isolation, dose adjustments and careful monitoring remain essential, and its use should be weighed against potentially safer alternatives (Paredes et al., 2020; Georgakis et al., 2025).

Current perioperative society guidelines offer only a limited population-specific direction here: the 2023 American Society of Anesthesiologists practice guidelines on neuromuscular monitoring and antagonism recommend quantitative monitoring to confirm adequate reversal in all patients but do not make separate recommendations for renal impairment (Thilen et al., 2023). Consistent with this evidence gap, the use of sugammadex product labeling in the US and Europe is advised against its use in patients with severe renal impairment, including those requiring dialysis (creatinine clearance <30 mL/min), because of insufficient safety data in this population (Merck Sharp and Dohme LLC, 2026; European Medicines Agency). Although sugammadex is nonetheless used in patients with end-stage renal disease, published clinical recommendations emphasize mandatory quantitative neuromuscular monitoring, preferential targeting of moderate rather than deep blocks when feasible, and, in dialysis-dependent patients, consideration of high-flux hemodialysis after surgery, since the sugammadex-rocuronium complex is removable by high-flux dialysis (Oh and Lim, 2023).

#### 5.4.6 Patients with liver failure

Rocuronium is eliminated mainly by the liver, and the recovery period of neuromuscular blockade in patients with liver failure is prolonged, while the plasma clearance rate in patients with cirrhosis is reduced (van Miert et al., 1997). The prolonged use of rocuronium may result in its accumulation in patients with liver failure and direct toxic effects. *In vitro*, rocuronium has also been shown to result in dose-dependent toxicity to the permanent human liver cell line, such as decreased cell viability, albumin synthesis, and cytochrome 1A2 activity (Sauer et al., 2017). Therefore, rocuronium is advised to be used with caution in patients with liver failure (Jain et al., 2026). Sugammadex is eliminated mainly by the kidney, and its use can lead to changes in the elimination of rocuronium. After being encapsulated by sugammadex, the plasma clearance rate of rocuronium is assimilated into the plasma clearance rate of sugammadex, and as the dose of sugammadex increases, the amount of rocuronium eliminated into urine also increases through the complex of sugammadex and rocuronium (Gijsenbergh et al., 2005), indicating that the use of sugammadex may contribute to reducing the potential liver toxicity caused by rocuronium.

A randomized controlled study revealed that in patients with child class “A” liver cirrhosis and normal liver function, the recovery times for TOFR >0.9 after postoperative administration of sugammadex were 3.1 min and 2.6 min, respectively (Abdulatif et al., 2018). Another study revealed that in patients with liver dysfunction, sugammadex can quickly and safely reverse neuromuscular blockade after continuous infusion of rocuronium (Fujita et al., 2014). However, no relevant studies have been conducted on sugammadex in patients with severe liver dysfunction. Therefore, its label still explicitly states that caution should be exercised when sugammadex is administered to patients with hepatic impairment accompanied by coagulopathy or severe edema (Merck Sharp and Dohme LLC, 2026).

#### 5.4.7 Others

Sugammadex effectively reversed neuromuscular blockade in various special patient populations, including patients with current or a history of pulmonary disease (e.g., asthma, chronic bronchitis, chronic obstructive pulmonary disease), cardiac disease (i.e., ischemic heart disease, chronic heart failure, arrhythmia), and hepatic dysfunction (liver damage class B or C) (Fujita et al., 2014; Amao et al., 2012; Dahl et al., 2009). For example, sugammadex two or 4 mg/kg was effective in patients with pulmonary disease, with a geometric mean time to recovery of the TOFR of 0.9 of 2.1 and 1.8 min, respectively (Amao et al., 2012). In addition, the reversal of neuromuscular blockade was ∼20 times faster with sugammadex two or 4 mg/kg than with placebo in patients with cardiac disease (Dahl et al., 2009).

## 6 Safety of sugammadex

Sugammadex has been found to be safe and well tolerated in most patients (Chandrasekhar et al., 2026). Compared with the traditional muscle relaxant antagonist neostigmine, sugammadex is relatively safer (Subramani et al., 2021; Kheterpal et al., 2020; Hristovska et al., 2017). A meta-analysis of sugammadex revealed a lower incidence of pulmonary complications after surgery than did neostigmine (Liu et al., 2023). Furthermore, in a clinical study, sugammadex was found to prevent postoperative urinary retention in minimal hernia surgery patients (Tsouknidas et al., 2024). Although the safety profile of sugammadex is more favorable than that of neostigmine, adverse reactions continue to be a matter of concern in its clinical use.

### 6.1 Residual neuromuscular blockade (RNMB) and recurarization

One of the major side effects of sugammadex is the possible recurrence of neuromuscular blockade despite the initial reversal of neuromuscular blockade (Iwasak et al., 2017; Dubois et al., 2023). An inadequate dose of sugammadex is thought to be the probable cause of RNMB or recurarization (Chandrasekhar et al., 2026).

RNMB is defined as a TOFR of less than 0.9 following the administration of neuromuscular blocking agent antagonists (Murphy and Brull, 2010). The reported incidence of RNMB varies substantially, which is attributable primarily to the use of neuromuscular monitoring and the time interval following sugammadex administration. In a study of 117 patients who received sugammadex without neuromuscular monitoring, the incidence of RNMB during spontaneous recovery in the post-anesthesia care unit (PACU) was found to be 4.3% (1.7%–9.4%) (Kotake et al., 2013). In another subsequent comparative study of 29 patients who received sugammadex with neuromuscular monitoring and 13 patients who received sugammadex without neuromuscular monitoring, the incidences of RNMB in the PACU were 0% and 15%, respectively (Ozbey et al., 2022). Furthermore, in a multicenter, observational study of 340 patients who received sugammadex with neuromuscular monitoring, the incidence of RNMB in the PACU was 5.3% (Table 4) (Esteves et al., 2023). A meta-analysis further revealed that the incidence of RNMB after sugammadex administration for moderate block was 19.2% at 2 min post-administration, 2.8% at 6 min post-administration, and 0.05%–2.8% at 10–60 min post-administration (Raval et al., 2020). With respect to deep block, the incidence of RNMB after sugammadex administration was 87.0% at 2 min post-administration, 44.7% at 4 min post-administration, 17.1% at 6 min post-administration, and 0.4%–11.3% at 10–60 min post-administration (Raval et al., 2020). Neuromuscular monitoring and time after administration are two important risk factors for RNMB after sugammadex administration.

**TABLE 4:** Incidence of RNMB (TOFR <0.9) after sugammadex administration in the PACU (Kotake et al., 2013; Ozbey et al., 2022; Esteves et al., 2023).

Parameters	Japanese (n = 117) (Kotake et al., 2013)	Portuguese (n = 340) (Esteves et al., 2023)	Turks (n = 29) (Ozbey et al., 2022)	Turks (n = 13) (Ozbey et al., 2022)
Incidence	4.3%	5.3%	0.0%	15.0%
Monitoring type	No	Acceleromyography	No	Acceleromyography
Sex-female	63.2%	56.6%	49.1%	48.9%
Age (years)	58	59	51	52
BMI (kg/m^2^)	21.7	26.4	27.7	28.6
ASA physical status I	44.4%	10.1%	83.4%	78.7%
ASA physical status II	55.6%	58.5%		
ASA physical status III	0%	30.60%	16.60%	21.30%

Data are given as the mean or median. BMI, body mass index; ASA, American Society of Anesthesiologists. in portuguese and turks, the parameters of sex, age, BMI, and ASA, are obtained from all enrolled patients.

Collectively, these data underscore that the utilization of neuromuscular monitoring and the elapsed time since sugammadex administration constitute two pivotal risk determinants for the occurrence of RNMB.

Recurarization is considered a reduction in the TOFR from ≥0.9 to <0.8 over three consecutive measurements (Duvaldestin et al., 2010; Loupec et al., 2016). The BRIDION prescribing information revealed that recurrence of neuromuscular blockade was a rare event in clinical trials, occurring in 0/895 (0%) patients receiving sugammadex 2 mg/kg, 1/1,921 (0.05%) receiving 4 mg/kg, and 2/98 (2%) receiving the higher 16 mg/kg dose (Merck Sharp and Dohme LLC, 2026). According to a dose-exploration study involving more than 2,000 patients, the incidence of recurarization after receiving the recommended dose of sugammadex is approximately 0.02% (Dubois et al., 2023). In Japan, from 2010 to 2018, 36 cases of recurarization resulting from sugammadex were reported, which led the safety committee of the Japanese Society of Anesthesiologists (JSA) to issue a warning in 2019 about the use of the correct dose of sugammadex (APSF, 2021). A study of three patients who underwent long-term tumor surgery revealed recurrence with a TOFR ≤0.6, possibly related to the accumulation of rocuronium which required reintubation (Katerenchuk et al., 2024; Li et al., 2023). A lower body mass index, elevated acute phase proteins, the use of magnesium sulfate, and rocuronium accumulation are considered risk factors for recurarization after NMBA antagonist administration (Dubois et al., 2023).

### 6.2 Hypersensitivity and anaphylaxis

Another major safety concern with sugammadex is the potential to induce hypersensitivity and anaphylactic reactions in a subset of patients (Zecic et al., 2022). Epidemiological data indicate considerable regional variation in incidence, with reported rates of 0.02% in Japan and 0.00016% in the United Kingdom (UK) (Savic et al., 2020). A retrospective analysis of phase 1–3 clinical trials suggested that the overall incidence of sugammadex-induced hypersensitivity is low (<1%) and comparable to that observed with placebo and neostigmine (Min et al., 2018a).

However, accumulating evidence indicates that the occurrence of such reactions is closely related to the dosing regimen. A recent meta-analysis revealed that while anaphylaxis following a single dose of sugammadex is rare, it can be severe and necessitates immediate intervention (Zecic et al., 2022). In contrast, repeated administration appears to be associated with a substantially higher incidence of hypersensitivity. For instance, one industry-funded study reported an incidence as high as 9.5% (Min et al., 2018b), and several studies have demonstrated a dose-dependent relationship (de Kam et al., 2018). Specifically, one clinical trial revealed that after three repeated doses of 4 mg/kg, the hypersensitivity rate was approximately 0.7%, whereas it was 4.7% in the 16 mg/kg group (de Kam et al., 2018). Another study with a similar repeated-dose design reported rates of 6.6% and 9.5% for the 4 mg/kg and 16 mg/kg groups, respectively (Table 5) (Min et al., 2018b).

**TABLE 5:** Incidence of hypersensitivity or anaphylaxis associated with sugammadex (de Kam et al., 2018; Takazawa et al., 2016; Harper et al., 2018; Miyazaki et al., 2018; Orihara et al., 2020).

Parameters	Incidence	Type	Dosage
US & Belgium (n = 299)* (Min et al., 2018b)	6.6% (10/151)	Hypersensitivity	4 mg/kg
	9.5% (14/148)	Hypersensitivity	16 mg/kg
Germany & Netherlands & UK & US (n = 298)* (de Kam et al., 2018)	0.7% (1/148)	Hypersensitivity	4 mg/kg
	4.7% (7/150)	Hypersensitivity	16 mg/kg
UK (n = 64121) (Harper et al., 2018)	0.0016% (1/64121)	Anaphylaxis	—
Japan (n = 3090000) (Takazawa et al., 2016)	0.0029% (29/1000000)	Anaphylaxis	—
Japan (n = 15479) (Miyazaki et al., 2018)	0.039% (6/15479)	Anaphylaxis	—
Japan (n = 29962) (Orihara et al., 2020)	0.02% (6/29962)	Anaphylaxis	—

* three repeat administrations.

The use of sugammadex in pediatric populations also remains a subject of debate. Although isolated case reports have attributed hypersensitivity reactions in children to sugammadex (Banoub et al., 2023; Choi and Yang, 2022), a large case-control study encompassing 835,405 pediatric patients revealed no statistically significant association between sugammadex exposure and anaphylaxis (Tadokoro et al., 2018). Furthermore, it is important to consider that some hypersensitivity reactions may be attributable to concurrently administered rocuronium rather than to sugammadex itself, and the increased concomitant use of these two agents may contribute to an increase in overall anaphylaxis rates (Takazawa et al., 2016).

### 6.3 Cardiovascular effects

Sugammadex has also been clinically evaluated for its cardiac effects and its potential to pose additional risks in patients with underlying cardiac conditions. A recent study of sugammadex in adults revealed that in 200 patients, bradycardia occurred approximately 4 min after sugammadex administration in 13 patients (6.5%) but was not associated with hemodynamic changes (Sims et al., 2020). A study of heart rate changes revealed that in 221 children, the incidence of bradycardia was 8.1%, and 38.9% of patients with bradycardia had comorbid cardiac conditions (Alsuhebani et al., 2020). Another study of heart rate changes in infants and children with heart disease reported that in 99 patients with a median age of 3 years, bradycardia was found in 20 patients (20.2%), but six patients were bradycardic prior to sugammadex administration (Table 6) (Arends et al., 2020). The association between the incidence of bradycardia and patients with cardiac complications seems to be controversial. A recent review revealed that bradycardia induced by sugammadex is common, ranging from 1% to 7%, and although most episodes do not necessitate therapeutic intervention, close monitoring is nonetheless recommended (Johnson and Chacin, 2023).

**TABLE 6:** Incidence of bradycardia associated with sugammadex (Sims et al., 2020; Alsuhebani et al., 2020; Arends et al., 2020).

Parameters	US (n = 200) (Sims et al., 2020)	US (n = 221) (Alsuhebani et al., 2020)	US (n = 99) (Arends et al., 2020)
Bradycardia	6.5% (13/200)	8.1% (18/221)	20.2% (20/99)
Age (years)	55	9	3
Bradycardia in patients with cardiac comorbidity	5.9% (2/34)	38.9% (7/18)	20.6% (20/97)
Occurring after administration	4 min	2 min	—
Dosage	4 mg/kg	2.1 mg/kg	3.9 mg/kg

Data from age, occurring after administration and dosage are given as median or mean.

### 6.4 Coagulation effects

With the increasing use of sugammadex in general anesthesia, it is important to assess its potential effects on coagulation, as this is a key safety concern for bleeding risk. Sugammadex was found to significantly affect various coagulation assays because of its ability to encapsulate phospholipids (Dirkmann et al., 2016). Further *in vitro* study of sugammadex with various anticoagulants revealed that sugammadex can increase activated partial thromboplastin time (aPTT) and prothrombin time (PT) under all anticoagulant conditions (Kruithof et al., 2020). Clinical studies have shown that although sugammadex resulted in a transient prolongation of aPTT and PT within minutes, these effects were typically short-lived and had no clinical significance (Rahe-Meyer et al., 2014; Raft et al., 2015). A recent large-scale meta-analysis (n = 1,328) indicated that sugammadex prolonged PT but not aPTT, suggesting that while routine monitoring was unnecessary in healthy patients, personalized evaluation remained advisable for high-risk individuals (Tsai et al., 2025). It is also critical to assess whether sugammadex-associated coagulation prolongation translates to increased surgical bleeding risk. One study revealed a greater postoperative bleeding volume in patients who received sugammadex than in those who received neostigmine (Tas et al., 2015). Despite initial concerns, extensive clinical evidences have emerged showing no correlation between sugammadex use and increased postoperative bleeding. For instance, a rigorously designed retrospective cohort study involving 29,062 patients revealed no statistically significant increase in bleeding risk associated with sugammadex administration (Schmidt et al., 2023).

## 7 Pharmacoeconomics of sugammadex

Cost is a further consideration relevant to the adoption of a neuromuscular blocking antagonist. Compared with neostigmine, sugammadex itself remains substantially more expensive per dose, with acquisition costs ranging from approximately 60-fold to more than 100-fold higher on a per-dose basis, depending on the depth of blockade (Paton et al., 2010). Pharmacoeconomic analyses have reached mixed conclusions, which primarily arise from differences in clinical benefit assessment, healthcare system cost structures, clinical scenarios, and dosing strategies (Carron et al., 2016; Fiorda Diaz et al., 2022; Lan et al., 2023).

Clinical benefit assessment: The value of operating room (OR) time saved is the key determinant. A UK systematic review and economic evaluation revealed that sugammadex is cost-effective if each OR minute saved exceeds £2.40 (moderate blockade) or £1.75 (deep blockade), with the OR time value (£4.44/min) far exceeding the PACU time (£0.33/min) (Paton et al., 2010). An Italian single-center study (128 cases) reported a net savings of €2.9 per case after accounting for time savings and additional drug costs (Carron et al., 2016).

Healthcare system cost structures: A propensity-matched study revealed that total costs were significantly higher in the sugammadex group than in the neostigmine group ($212.0 vs. $50.6) despite faster recovery and less bradycardia, which was attributed to low local labor costs (Lan et al., 2023).

Clinical scenarios: In CICV events, high-dose sugammadex (16 mg/kg) would be cost-effective compared with succinylcholine for very short procedures if it prevents one additional death per ∼1,979 uses in a 60-year-old patient (assuming zero CICV risk) at a willingness-to-pay of £20,000 per quality-adjusted life year (Chambers et al., 2010). The economic case is weaker for short or low-complexity procedures.

Dosing strategies: A randomized controlled trial revealed that sugammadex 2 mg/kg + neostigmine 50 μg/kg was non-inferior to sugammadex 4 mg/kg for deep blockade reversal (intention-to-treat: 96% vs. 89%; per-protocol: 100% vs. 92%) (O’Brien et al., 2017), suggesting that a reduced-dose regimen is a cost-saving alternative.

## 8 Perspectives on novel NMBA antagonists

In addition to the two marketed agents, neostigmine and sugammadex, several novel reversal agents are under investigation. Among them, amogammadex (also known as adamgammadex) is the most advanced, having completed phase 3 clinical studies and has been approved for marketing in China (Zhang et al., 2024). Other emerging agents, including calabadions, L-cysteine adduction, and cucurbit[8]uril derivatives, are still in the early stages of development and lack sufficient clinical validation (de Boer and Vieira Carlos, 2025).

Amogammadex, whose structure and encapsulation mechanism are similar to those of sugammadex (Hunter and Sneyd, 2024), was synthesized by conjugating N-acetyl-L-cysteine to each sugar unit of γ-cyclodextrin, resulting in at least a 20% increase in chiral carbon atoms and a substantial improvement in molecular binding selectivity (Figure 3) (Chen et al., 2015; Jiang et al., 2020). In terms of pharmacology, amogammadex can also rapidly reverse both moderate and deep neuromuscular blockade induced by rocuronium within 3 min (moderate antagonism for 2.25 min and deep antagonism for 2.48 min) (Zhang et al., 2024; Zhao et al., 2024). In terms of safety profiles, at the 4 mg/kg dose for moderate neuromuscular blockade, amogammadex (n = 155) possibly resulted in lower incidences of the special adverse events of interest related to sugammadex (n = 155): anaphylactic reaction (0% vs. 1.1%), recurarization (0% vs. 1.1%), decreased heart rate (1.9% vs. 3.2%), and laryngospasm (0% vs. 0.6%) (Zhang et al., 2024). However, given the relatively limited sample size in randomized controlled trials for amogammadex, whether this safety advantage can be further validated in real-world studies remains to be determined.

**FIGURE 3:**
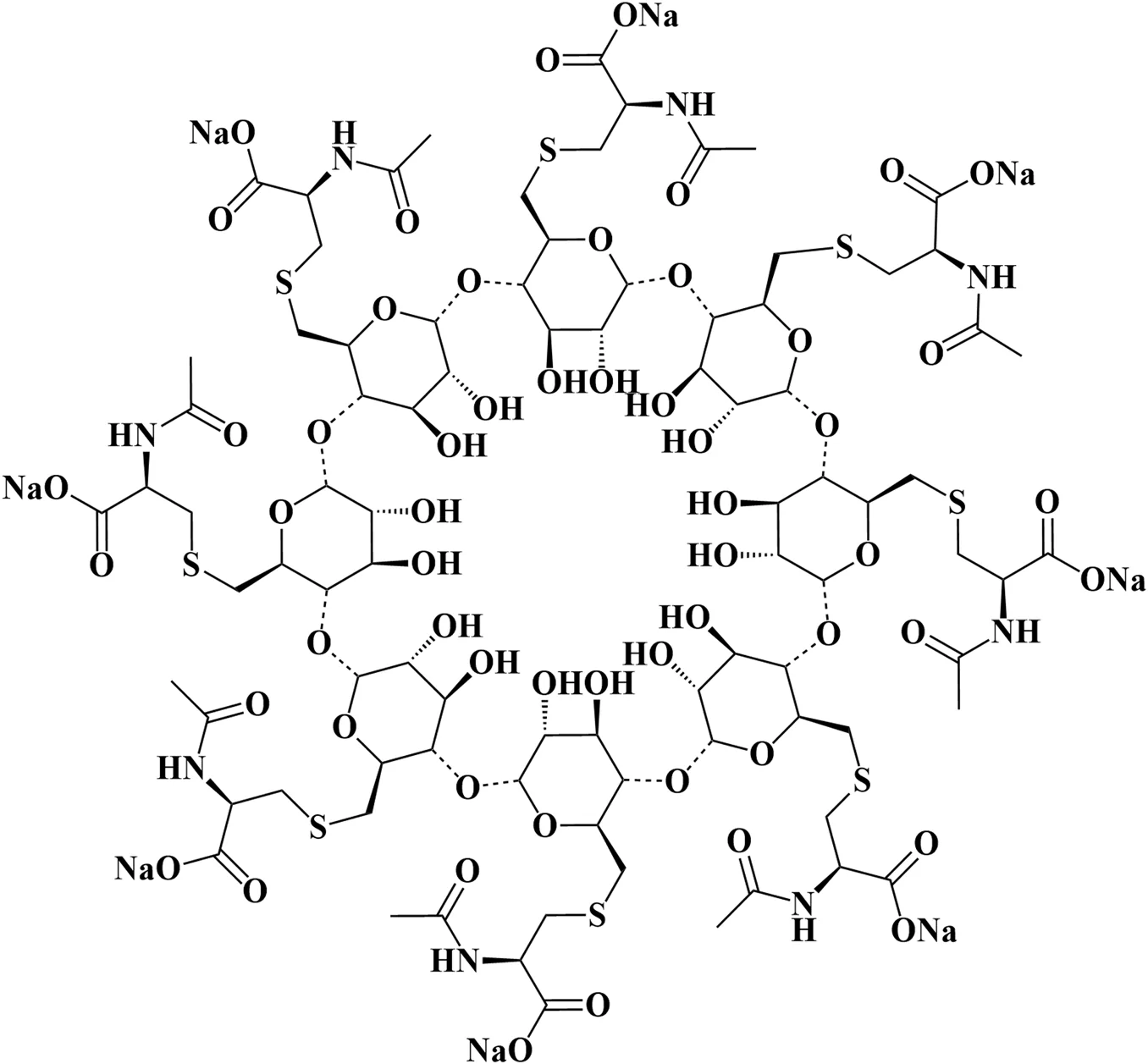
Chemical structure diagram showing amogammadex with eight side chains modified with N-acetyl-L-cysteine.

Calabadion (Calabadion one and Calabadion 2) is a cucurbituril-based synthetic molecule capable of simultaneously reversing both aminosteroidal (rocuronium and vecuronium) and benzylisoquinolinium (cisatracurium) NMBAs. It rapidly sequesters free relaxant molecules in plasma by forming host-guest complexes with them, thereby reducing their effective concentration at the neuromuscular junction and restoring normal neuromuscular conduction within tens of seconds (Haerter et al., 2015).

L-cysteine is an endogenous thiol-containing amino acid that selectively inactivates fumarate-based nondepolarizing neuromuscular blockers (e.g., gantacurium, CW002, CW011, and CW1759-50). Its mechanism involves covalent addition to the carbon-carbon double bond of these drugs, generating a stable thioether adduct that blocks their interaction with acetylcholine receptors. However, these NMBAs are still at an early developmental stage (Radkowski et al., 2026; Savarese et al., 2010).

A recently discovered cucurbit[8]uril derivative functions as a broad-spectrum reversal agent for neuromuscular block induced by both aminosteroidal and benzylisoquinolinium NMBAs. Its mechanism involves supramolecular sequestration, effectively encapsulating neuromuscular blocker molecules and facilitating their removal from the neuromuscular junction, thereby reversing the blockade (Liu et al., 2022).

## 9 Conclusion

As the first γ-cyclodextrin-based supramolecular agent successfully translated into clinical practice, sugammadex operates through a mechanism fundamentally distinct from that of traditional acetylcholinesterase inhibitors. Rather than modulating cholinergic transmission, sugammadex directly targets NMBAs such as rocuronium and vecuronium, which encapsulate them within its hydrophobic cavity to form inactive complexes. In terms of efficacy, compared with traditional antagonists, sugammadex dramatically shortened neuromuscular function recovery time from approximately 10 min or longer to within 3 min, enabling near “on-switch” reversal of blockade. This not only enhances operating room efficiency but also overcomes the ceiling effect that historically limited the reversal of deep neuromuscular blockade. Although adverse effects, including RNMB, recurarization, hypersensitivity, coagulation alterations, and cardiovascular effects, have been observed in a very small subset of patients, these effects have not impeded its widespread clinical adoption. Further reduction of these adverse effects and the development of broad-spectrum reversal agents may represent directions for next-generation antagonist research.

## 10 Limitations

This review has several limitations. As a narrative rather than a systematic review, the literature was synthesized qualitatively without formal quality appraisal or risk-of-bias grading; selection and citation bias cannot be fully excluded, although we have endeavored to present conflicting findings and to indicate the strength of evidence underpinning individual claims. It should also be noted that most of the authors are employees of Hangzhou Adamerck Pharmlabs Inc., which is developing amogammadex, a competitive agent to sugammadex (Section 8), and readers should bear this in mind when interpreting the content of this review.
